# De-epithelialized overlap flap to secure urethroplasty in second stage hypospadias repair: revisiting the Smith technique

**DOI:** 10.1186/s12894-023-01312-8

**Published:** 2023-08-30

**Authors:** Amr Abdelhamid AbouZeid, Reda Abualyazeed Habak, Mostafa Mahmoud Hamad, Alaa-Eldin Medhat Shahin

**Affiliations:** 1 Department of Pediatric Surgery, Faculty of medicine-Ain Shams University, Cairo, Egypt; 2 Department of Pediatric Surgery, Benha Specialized Children Hospital, Benha, Egypt

**Keywords:** Proximal hypospadias, Curvature, Second layer, Chordee, Urethral plate, Two-stage repair

## Abstract

**Background:**

The application of a second layer between the neourethra and skin was a major contribution, which has improved the outcome of hypospadias repair. Here, we report our experience of revisiting the original Smith technique using a de-epithelialized overlap flap to support the urethroplasty in staged hypospadias repair.

**Methods:**

The study included primary cases of proximal hypospadias with significant chordee who underwent two-stage repair during the period 2016 through 2021. The ventral curvature was corrected at first stage by excision of the urethral plate, followed by covering the ventral shaft by skin flaps or inner preputial graft. The second stage (Thiersch -Duplay urethroplasty) was performed six months later. The de-epithelialized overlap flap (double breasting) technique was used to cover the neo-urethra in all cases, which was combined with a dartos scrotal flap to cover the proximal neourethra when indicated.

**Results:**

The study included 17 boys with proximal hypospadias who underwent two-stage repair. Follow up period after the second stage ranged between 6 and 30 months (mean 19.7; median 18.5). Post-operative complications were detected in 7 cases (41%). Most complications were related to distal/glanular disruptions whether partial or complete (5 cases). One case developed a penoscrotal fistula that was closed surgically. Another case (belonging to the group which used preputial graft in the 1st stage) presented 21 months after the second stage with urethral stricture (penoscrotal).

**Conclusion:**

Applying the de-epithelialized double-breasting skin closure can offer alternative second layer coverage for the neourethra along the penile shaft in staged repair of proximal hypospadias.

## Background

While surgery for distal hypospadias is considered an aesthetic procedure, surgery for proximal hypospadias aims to improve function [1]. Two main goals should be achieved after successful repair for a proximal hypospadias: to urinate freely (without strictures/stenosis) through a distal meatus, and to correct associated ventral curvature [2, 3]. In the 1990s, experts argued that ventral curvature was not caused by the urethral plate, and that it should be preserved as much as possible. [4]. However, this concept failed the test of time when several centres all over the world reported recurrence of chordee after plate-preserving techniques for proximal hypospadias. Recently, there has been an obvious worldwide trend back to two-stage repairs for proximal hypospadias [5–8]. The urethral plate is excised in the first stage and substituted by skin flaps or grafts, to be tubularized six months later at the second stage (Thiersch-Duplay urethroplasty).

We can all agree that adding a second layer between the neourethra and skin had a significant impact on the success of the hypospadias correction [9]. This development may be attributed to Smith’s use of his de-epithelialized double-breasting skin closure technique in the 1970s [10], although Pers had earlier detailed the similar concept of skin shaving in 1965 [11]. Retik et al. described using dorsal subcutaneous flap as a second layer to prevent urethro-cutaneous fistula [12, 13], which gained widespread acceptance especially with distal and mid-penile hypospadias. With more severe types of hypospadias, the tunica vaginalis flap (introduced by Snow) [14] has become the preferred second layer coverage in many centers compensating for associated shortage of penile skin that is usually spared for the urethroplasty.

Here, we report our experience of revisiting the original Smith technique using a de-epithelialized overlap flap to support the urethroplasty in staged repair of proximal hypospadias.

## Methods

We retrospectively analysed the data of primary cases of proximal hypospadias with significant chordee who underwent two-stage repair during the period 2016 through 2021. Only cases who applied the de-epithelialized double breasting skin closure at second stage of repair were included in this study.

### Surgical technique

**Fig. 1:**
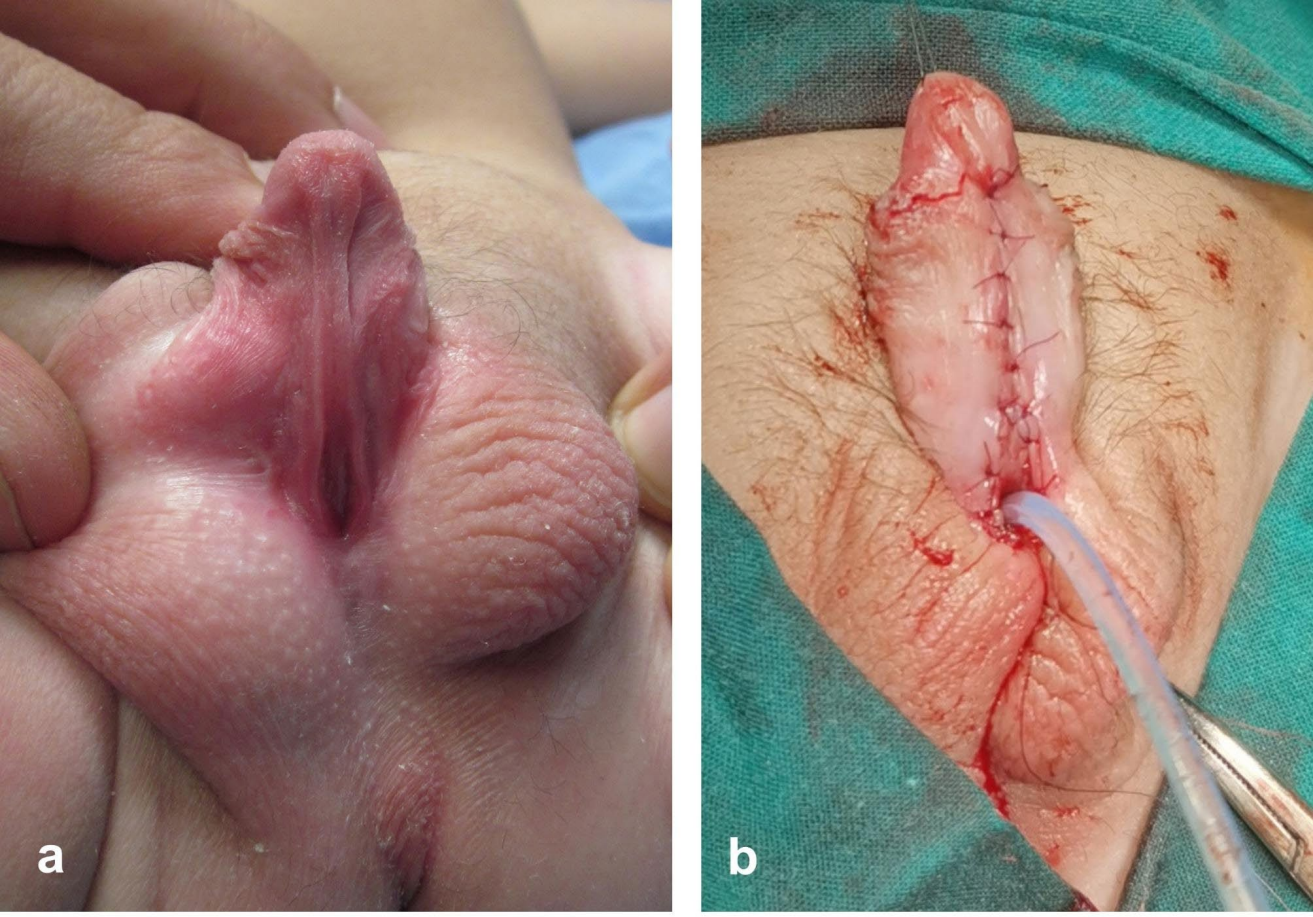
First-stage of hypospadias repair: **(a)** Eight-month-old boy with scrotal hypospadias; **(b)** excision of urethral plate to straighten the penis, while the penile ventrum was covered by skin (Byars) flaps

**Fig. 2:**
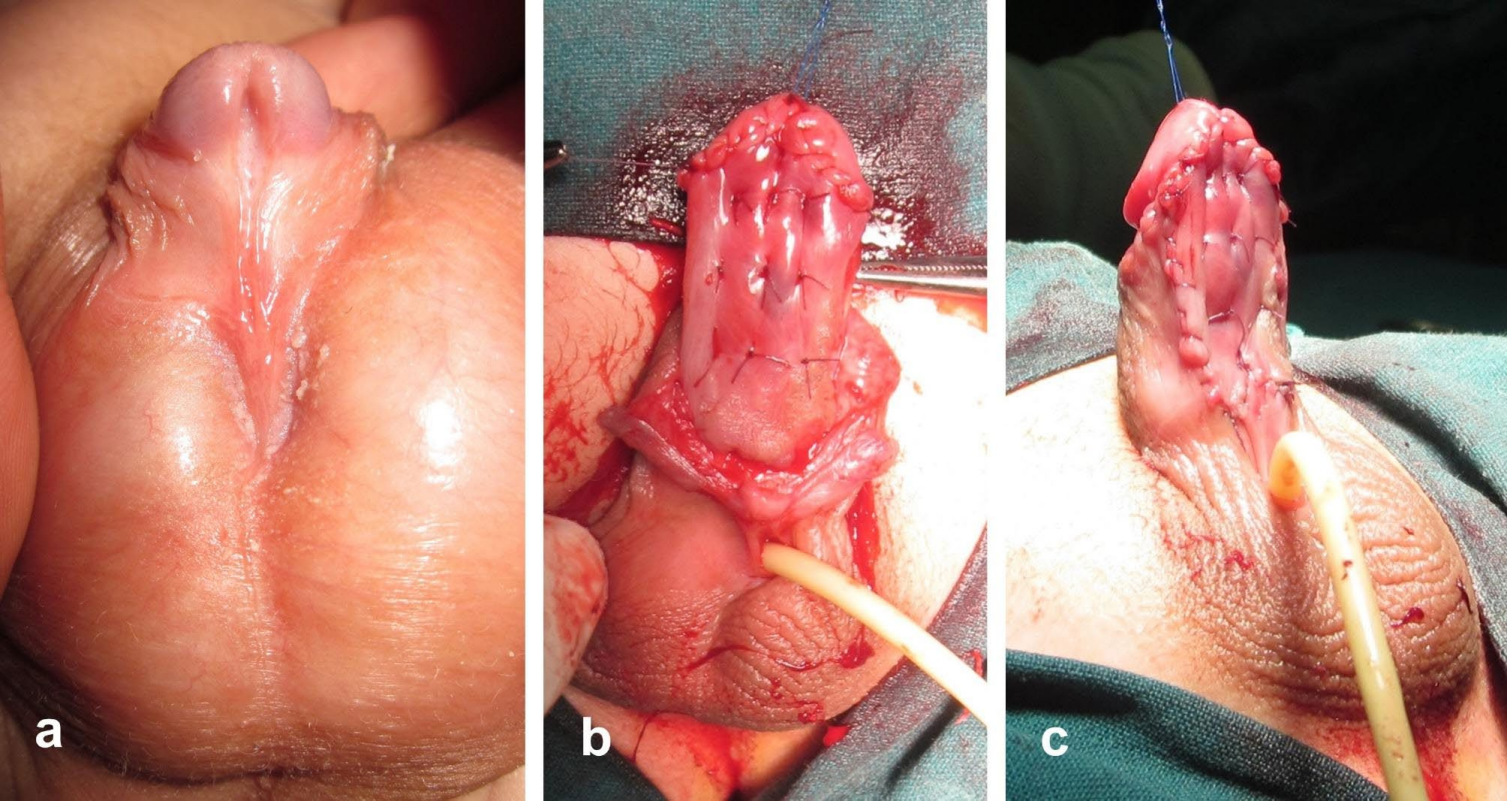
First-stage of hypospadias repair: **a)** Ten-month-old boy with peno-scrotal hypospadias; **(b** and **c)** excision of urethral plate to straighten the penis, while the penile ventrum was covered by inner preputial graft

The first stage starts by degloving of the penile skin. Ventral dissection is extended down to the scrotum exposing the bulbar urethra. The position of the hypospadiac meatus as well as the site of bifurcation of the corpus spongiosum is defined after complete degloving reflecting the degree of severity of hypospadias. In presence of significant ventral curvature (chordee > 30°), the decision is made to sacrifice the urethral plate. The urethral plate together with all subjacent dysplastic tissue are meticulously excised to straighten the penile shaft. This will relatively shift the meatus to a more proximal position. Either skin flaps (Fig. 1) or inner preputial graft (Fig. 2) is used to cover the ventral surface of the penile shaft; this represents a substitute for the urethral plate to be tubularized six months later at the second stage.

**Fig. 3:**
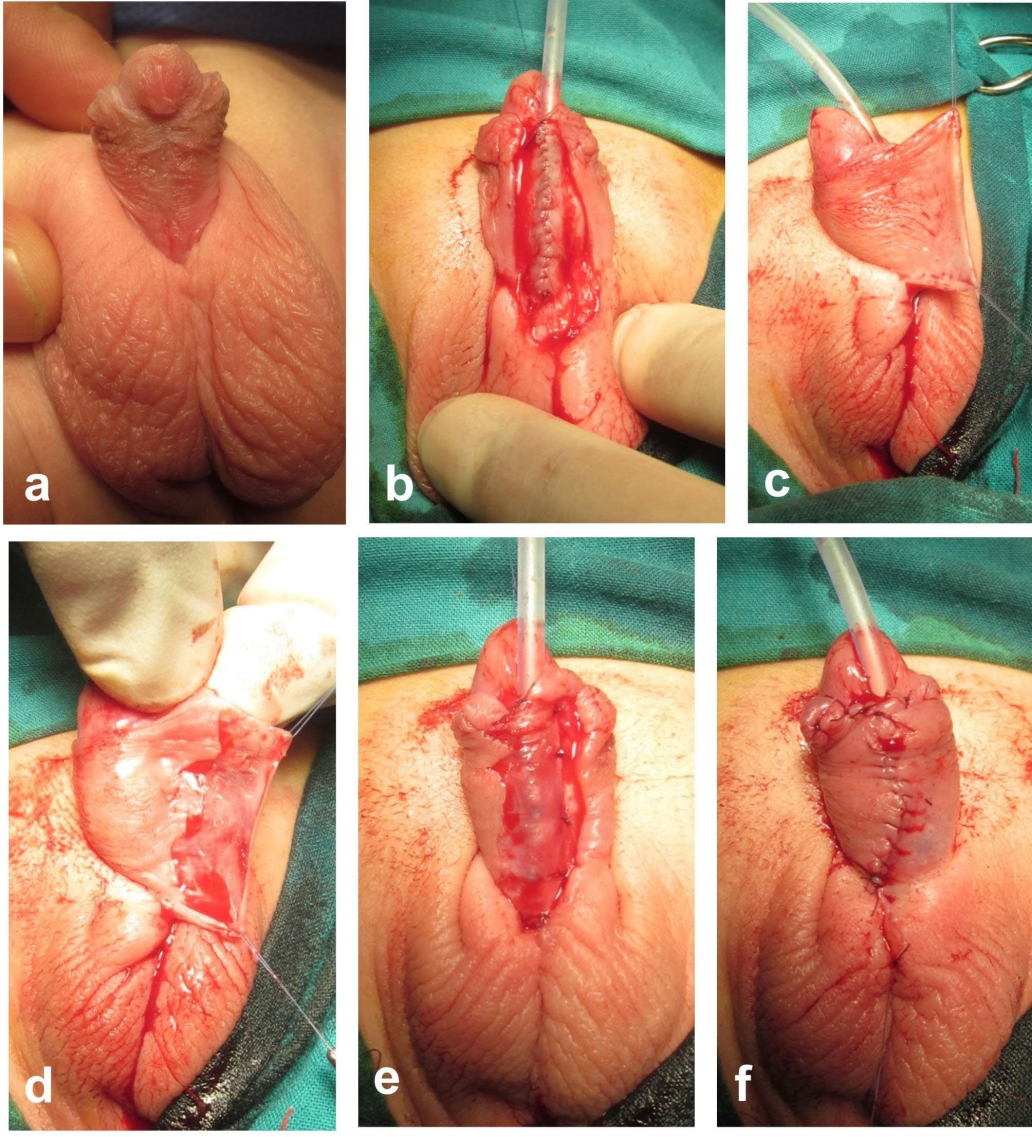
Second-stage of hypospadias repair (same case in Fig. 1, six months later). **(a)** The penis after correction of chordee in the 1st stage. **(b)** Thiersch-Duplay urethroplasty. **(c** and **d)** A rectangular area is de-epithelialized along the free edge of the penile skin on one side. **(e** and **f)** The de-epithelialized skin flap is fixed to the penile shaft over the neo-urethra to be overlapped by the skin flap from the other side (double breasting skin closure)

**Fig. 4:**
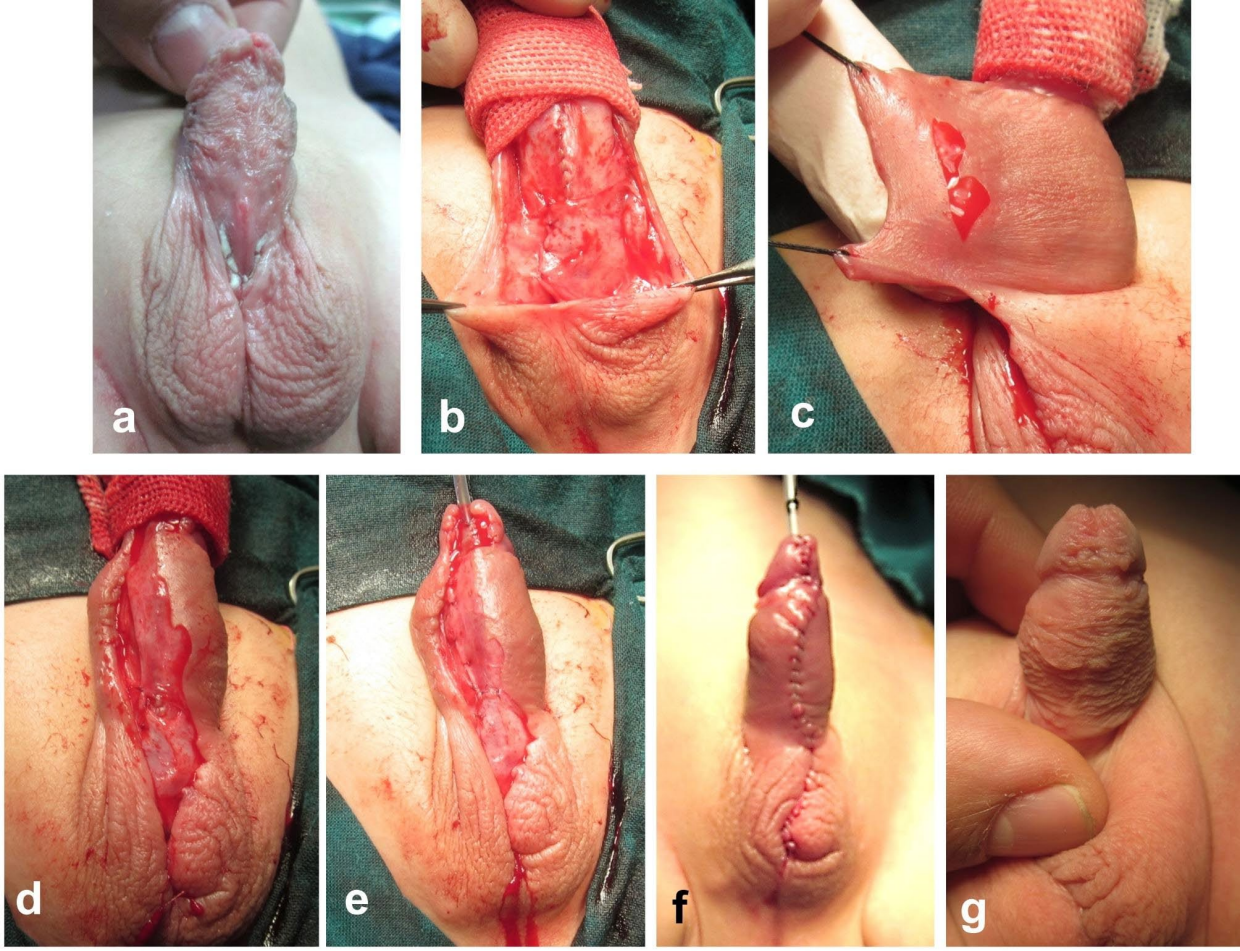
Second-stage of hypospadias repair (same case in Fig. 2, six months later). **(a)** The penis after correction of chordee in the 1st stage. **(b)** Thiersch-Duplay urethroplasty; the proximal end of the neourethra is covered by scrotal dartos flap. **(c** and **d)** A rectangular area is de-epithelialized along the free edge of the penile skin on one side. **(e** and **f)** The de-epithelialized skin flap is fixed to the penile shaft over the neo-urethra to be overlapped by the skin flap from the other side (double breasting skin closure). **g)** The penile appearance one year later after second stage; note the presence of glanular fistula

During the second stage, a Thiersch-Duplay urethroplasty is applied to reconstruct the anterior urethra transferring the urinary meatus distally to the glans. The urethroplasty is performed using polyglactin 6 − 0 continuous (full thickness) suturing technique; a second layer of interrupted sutures are applied at wide intervals to support the underlying continuous suture line of the urethroplasty. Before skin closure, a rectangular area is de-epithelialized along the free edge of the penile skin on one side as shown in Fig. 3. The de-epithelialized skin flap is fixed to the penile shaft over the neo-urethra to be overlapped by the skin flap from the other side (double breasting skin closure). For cases with extra-long urethroplasties (scrotal/perineal hypospadias), a scrotal dartos flap is used to cover the proximal neourethra, while the de-epithelialized overlap flap (Smith technique) is used to cover the rest of neo-urethra (Fig. 4).

## Results

The study included 17 boys with proximal hypospadias who underwent two-stage repair. In all cases, the de-epithelialized overlap flap (double breasting) technique was used to cover the neo-urethra, which was combined with a dartos scrotal flap to cover the proximal neourethra when indicated. Their age at first stage ranged between 6 and 66 months (mean 23, median 21). The meatus position was penoscrotal in eight, scrotal in seven, and perineal in two. In all cases, the ventral curvature was corrected at first stage by careful dissection and excision of the urethral plate with all subjacent dysplastic tissues on the penile ventrum (neither ventral corporotomies, nor dorsal plication were applied). Dorsal skin flaps were used to substitute the urethral plate in 10 cases, while inner preputial graft was used in the rest (7 cases). No complications were detected after the first stage in this case series. The second stage (Thiersch -Duplay urethroplasty) was performed at least six months after the first stage.

**Fig. 5:**
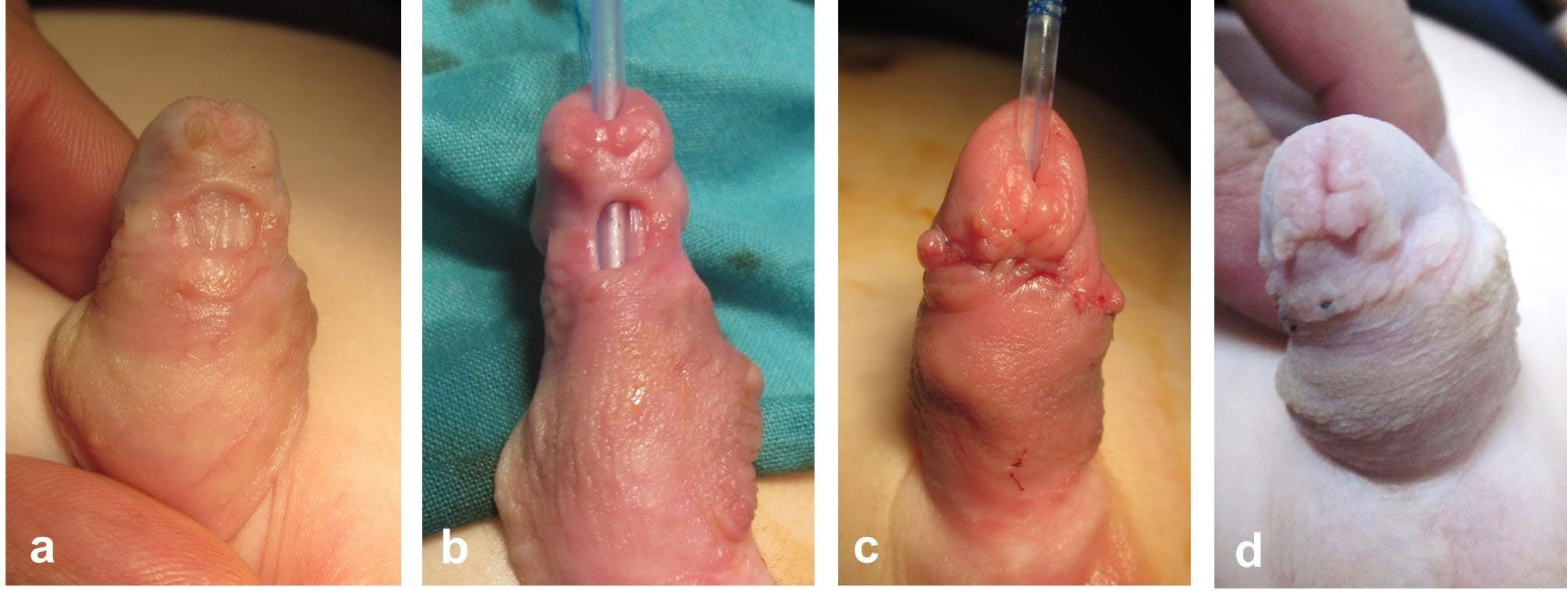
**(a** and **b)** Large coronal fistula after two-stage hypospadias repair. **c)** Closure of fistula. **d)** Penile appearance two years later at follow-up

**Fig. 6:**
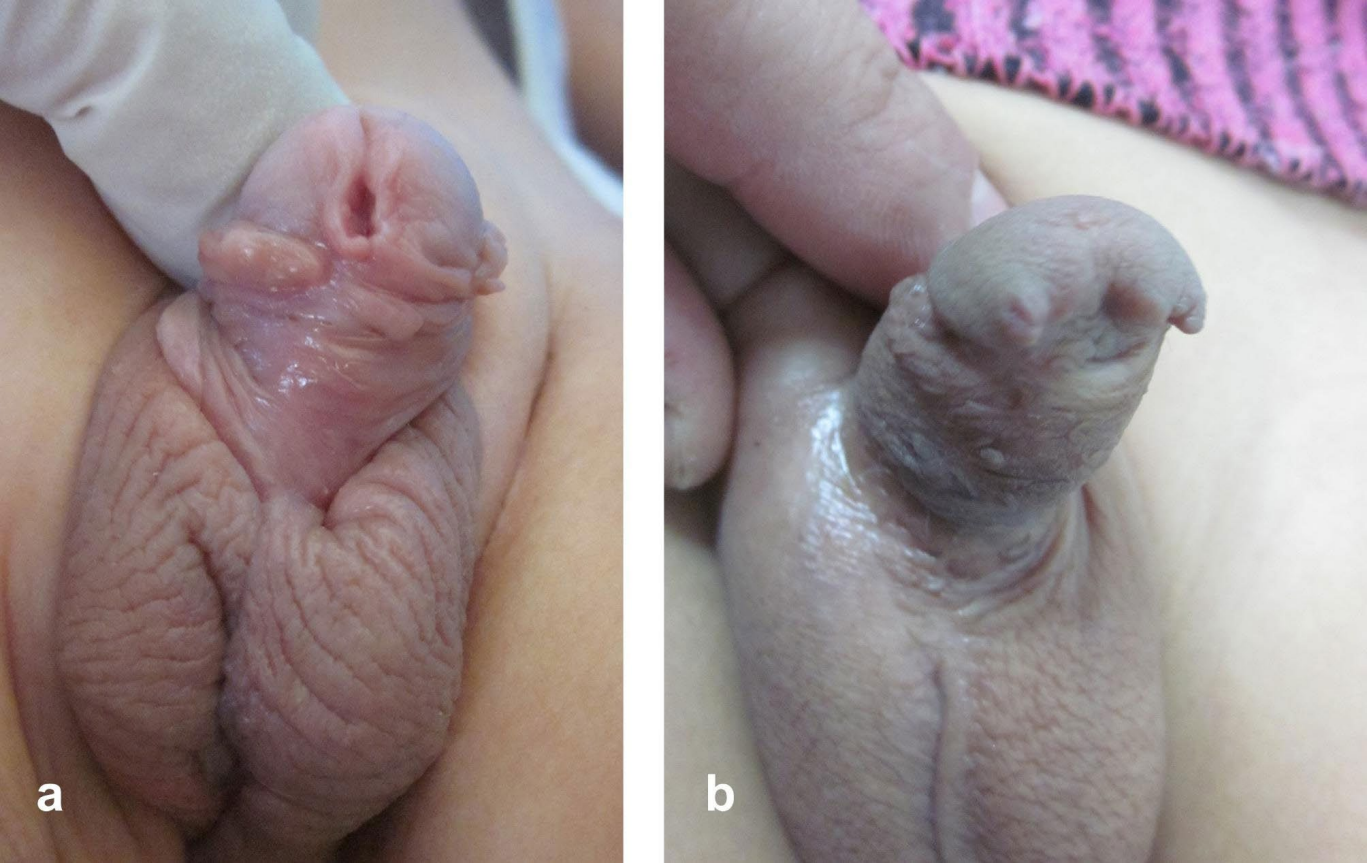
Penile appearance at follow up following 2nd stage repair in two different cases of proximal hypospadias: **(a)** without glanular dehiscence; **(b)** with glanular dehiscence. Note the subglanular position of the neo-meatus in **(b)**, which may be considered both functionally and cosmetically acceptable.a

Follow up period after the second stage ranged between 6 and 30 months (mean 19.7; median 18.5). Post-operative complications were detected in 7 cases (41%); six of them required a third intervention (Table 1). Most complications were related to distal/glanular disruptions whether complete glanular dehiscence (3 cases) or partial dehiscence (fistula in 2 cases). A large coronal fistula was successfully closed surgically (Fig. 5); while in another case, a small glanular fistula was incised distally through the meatus to create a single opening (Fig. 4g). Another case developed a penoscrotal fistula that was also closed surgically; the latter complication was before we have started to use additional scrotal dartos flap to cover the proximal end of neourethra. Isolated glanular dehiscence (1 case) with coronal or sub-coronal meatus was considered both functionally and cosmetically satisfactory with no intention for reoperation (Fig. 6). A single case (belonging to the group which used preputial graft in the 1st stage) presented 21 months after the second stage with urethral stricture (penoscrotal). Trial of dilatation was unsuccessful, which was followed by a long urethrotomy down to the stricture. This case is being prepared for reoperation (staged repair).

**Table 1:** Postoperative complications following 2-stage repair for proximal hypospadias using Smith De-epithelialized overlap flap to secure urethroplasty

Study group (17 cases)	Complications		
	Disruption of distal neo-urethra	Urethro-cutaneous fistula	Urethral stricture
Inner preputial graft in 1st stage (7 cases)	2	2	1
Skin flaps in 1st stage (10 cases)	1	1	0
Total	3 (17.6%)	3 (17.6%)	1 (5.8%)

## Discussion

The interposition of a layer between the neourethra and the skin suture line represents one of the major technical advances in hypospadias surgery [9]. This principle has been introduced by Smith in 1973, when he described a de-epithelialized overlap flap to cover the neourethra in staged repair [10]. In his original report [10], Smith referred to his idea of skin shaving being previously described by Pers and Crawford [11]. The advantage of this overlap technique is that it allows for “tissue adhesion over a wide area rather than edge to edge healing” and without superimposed suture lines [10]. Currently, the dorsal dartos and the tunica vaginalis flaps (introduced later by Retik, and Snow, respectively) are the two more popular alternatives to cover the neourethra with different techniques of hypospadias repair [12–15].

In this report, we have revisited the original Smith technique [10], which we have found very suitable with the two-stage hypospadias repair. The de-epithelialized double-breasting skin closure offered excellent healing and support for the neourethra along the penile shaft, while complications were mainly restricted to the most distal (glanular) part. Although glanular dehiscence (partial or complete) was a common complication in this series, yet some experts may still consider subglanular/coronal meatus a success after proximal hypospadias repair [16], which can be both functional and cosmetically accepted [5]. We believe that multiple factors may be responsible for failure of glanular closure in patients with proximal hypospadias; the small size of the glans may be a major contributing factor. Moreover, the applied technique of de-epithelialized double-breasting skin closure may not be always sufficient to cover the whole length of the neo-urethra, especially at both ends. The proximal end of the neo-urethra can be covered by a nearby (readily available) scrotal dartos flap; however, the distal (glanular) end of the neourethra may be left unprotected by a second layer.

Proximal hypospadias represents a persistent challenge with no consensus on the best type of repair [17]. Preservation of the urethral plate seems to play a major role in recurrence of ventral curvature that may be under-reported [5, 17]. Over the past years, a lower threshold to sacrifice the plate and stage the repair has been observed [19]. Flaps versus grafts in two stage repair remains a controversial debate in the literature [18]. A major concern about grafts is liability for contracture and shrinkage even after years [18]. In this report, we had a complication of urethral stricture in one case from the ‘graft’ group. However, discussing this major complication is beyond our scope in this report, and will be studied in a separate one.

The two-stage hypospadias repair was the standard in the 1960 and 1970 s [5]. Later, in the 1980s, Duckett induced worldwide shift to one-stage repair by introducing the preputial island flaps [19]. The universal acceptance of the new concepts might have masked the success of the Smith’s de-epithelialized overlap flap. In 1988, Belman renewed the interest in the de-epithelialized flap applying it with modern techniques for hypospadias repair [20]; however, the dartos and tunica flaps were already taking over as more popular techniques for covering the neourethra. With the worldwide trend back to two-stage repairs [8], the Smith technique may be recalled to life.

The study is limited by the small sample size and lack of comparative group. A relatively high rate of complications (41%) was found, which may be quite expected in such group of severe hypospadias [8, 17]. When utilising tunica vaginalis as a second layer to cover urethroplasty in two-stage repair, Badawy and colleagues [21] observed a similarly high percentage of distal disruptions and fistulae (46%) but Snodgrass and Bush reported a lower rate (23%) [22]. In our experience, the Smith technique is much simpler than the tunica vaginalis flap. The latter is more invasive by dissection around the testis and is liable for contractures. Covering the neourethra by an intervening layer is not just for waterproofing [15], but also it is important to promote healing by providing neovascularity and growth factors [20]. This may be best achieved by using the subcutaneous tissue as a second layer for covering the neourethra. However, the Smith technique is mostly applicable for primary cases with abundant local skin, and maybe impractical in redo cases with deficient skin.

## Conclusion

With the trend back to two-stage repairs for proximal hypospadias, applying the de-epithelialized double-breasting skin closure is an alternative way to provide second layer coverage for the neourethra along the penile shaft.

## Data Availability

The datasets used and/or analyzed during the current study are available from the corresponding author on reasonable request.
